# *Lgr5* Identifies Progenitor Cells Capable of Taste Bud Regeneration after Injury

**DOI:** 10.1371/journal.pone.0066314

**Published:** 2013-06-18

**Authors:** Norifumi Takeda, Rajan Jain, Deqiang Li, Li Li, Min Min Lu, Jonathan A. Epstein

**Affiliations:** 1 Department of Cell and Developmental Biology, Perelman School of Medicine at the University of Pennsylvania, Pennsylvania, United States of America; 2 Penn Cardiovascular Institute, Perelman School of Medicine at the University of Pennsylvania, Pennsylvania, United States of America; 3 Institute of Regenerative Medicine, Perelman School of Medicine at the University of Pennsylvania, Pennsylvania, United States of America; Brigham & Women's Hospital - Harvard Medical School, United States of America

## Abstract

Taste buds are composed of a variety of taste receptor cell types that develop from tongue epithelium and are regularly replenished under normal homeostatic conditions as well as after injury. The characteristics of cells that give rise to regenerating taste buds are poorly understood. Recent studies have suggested that *Lgr5* (leucine-rich repeat-containing G-protein coupled receptor 5) identifies taste bud stem cells that contribute to homeostatic regeneration in adult circumvallate and foliate taste papillae, which are located in the posterior region of the tongue. Taste papillae in the adult anterior region of the tongue do not express *Lgr5.* Here, we confirm and extend these studies by demonstrating that *Lgr5* cells give rise to both anterior and posterior taste buds during development, and are capable of regenerating posterior taste buds after injury induced by glossopharyngeal nerve transection.

## Introduction

Taste buds are sensory organs detecting sweet, bitter, sour, salty, and umami (savory), and they are composed of at least three types of fusiform gustatory receptor cells (type I–III) as well as round cells in the basal compartment (type IV). Taste receptor cells in the adult are postmitotic and trophically maintained by the activity of taste neurons. They are a relatively short-lived and heterogeneous population of cells that are continually replaced by progenitor cells, the characteristics of which remain poorly defined [1]–[3].

Taste buds mature peri-natally within epithelial appendages, termed taste papillae, which arise at mid-gestation as epithelial thickenings or placodes. Recent lineage tracing experiments using inducible Cre-Lox technologies demonstrated that *Sonic hedgehog* (*Shh*)-expressing cells in the embryonic placodes are taste receptor cell progenitors, giving rise to mature taste buds in young postnatal animals [4]. However, these derivatives gradually disappear by 4 months of age, suggesting that additional, *Shh^-^*, progenitor cells are recruited to replenish the mature taste bud lineages in adult mice. Type IV cells have been proposed as a precursor cell population of taste receptor cells [2], [5], but they include a heterogeneous population that includes *Shh* and *Prox1*-expressing cells [6], [7]. In addition, fate-mapping studies of adult type IV cells have not been undertaken to confirm or refute this hypothesis.

There is some evidence suggesting that adult taste buds are derived from the surrounding extragemmal epithelium (i.e. epithelial progenitors extrinsic to the taste bud itself) [8], [9]. For example, cell lineage analysis using an X-linked mosaic transgenic mouse line has suggested that taste buds and the adjacent epithelial cells arise from a common progenitor residing within the local surrounding epithelium [8]. In addition, *Keratin 14* (*K14*)-expressing basal epithelial keratinocytes, genetically labeled using a Cre-Lox strategy, can give rise to taste receptor cells and to tongue epithelium [9]. Indeed, previous studies have suggested that taste papillae epithelial cells immediately surrounding taste buds are distinct from more remote tongue epithelial cells; they express high levels of Sox2 [10] and Shh target genes, *Patched* (*Ptc*) and *Gli1* [6], [11]. Thus, local surrounding epithelium represents a potential source of mature taste bud stem/progenitor cells, though experimental evidence for this hypothesis remains lacking.

*Lgr5* is expressed in stem/progenitor cells of multiple tissues, including the hair follicle and intestinal crypt [12], [13] and functions as a Wnt co-receptor in the β-catenin signaling pathway [14]. *Lgr5*-expressing cells have recently been shown to give rise to at least some new taste buds during the normal cycles of growth and regression [15]. Although *Lgr5* is detected in the tongue epithelium from embryonic stages [16] and Wnt/β-catenin function is necessary and sufficient for taste placode formation [17], [18], the role of *Lgr5^+^* cells during development of taste buds or after injury remains poorly elucidated. Here, we demonstrate that *Lgr5^+^* cells function as progenitor cells for taste buds during development. In addition, we show that *Lgr5^+^* cells can give rise to newly regenerated taste buds in the posterior region of the tongue during normal homeostasis and after injury in adult mice.

## Materials and Methods

### Mice

*Lgr5^EGFP-ERCre/+^* [12], *Shh^ERCre/+^* [19], *R26^Tom/+^* [20] mice have been described previously. All mice were maintained on mixed genetic backgrounds. The University of Pennsylvania Institutional Animal Care and Use Committee approved all animal protocols (Permit Number; 803396).

### Lineage Tracing Experiments

Mice were injected intraperitoneally with tamoxifen (100 mg/kg body weight; Sigma) dissolved in corn oil, as a single dose or daily for 5 consecutive days, as indicated.

### Glossopharyngeal Neurectomy (GLx)

Mice were intraperitoneally anesthetized with 2,2,2-tribromoethanol (300 mg/kg body weight; Sigma), and all efforts were made to minimize suffering. An incision was made along the ventral neck midline and the digastric muscle was exposed. The posterior belly of the digastric muscle was retracted, and the glossopharyngeal nerve passing between carotid arteries was transected as it entered the jugular foramen. Bilateral glossopharyngeal nerves were transected because taste buds of the circumvallate papilla are bilaterally innervated.

### BrdU Labeling Experiments

*In vivo* bromodeoxyuridine (BrdU) incorporation was performed to analyze cell proliferation. Mice were subjected to GLx, and 2 days later injected intraperitoneally with BrdU (100 mg/kg; Sigma). Tissues were collected 2 hours after BrdU administration, and then stained with an anti-BrdU monoclonal antibody (Rat, 1∶20, Accurate).

### Histology and Microscope

Tongues were fixed in 2% paraformaldehyde, ethanol dehydrated, embedded in paraffin, and sectioned. Antibodies used were: GFP (goat, 1∶100, Abcam), RFP (recognizes tdTomato) (rabbit, 1∶50, Rockland), Ki67 (rabbit, 1∶50, Santa Cruz), PCNA (mouse, 1∶50, Biocare), Sox2 (goat, 1∶10, Santa Cruz) (rabbit, 1∶500, Seven Hills Bioreagents), Phospholipase C, β2 (PLC β2) (goat, 1∶25, Santa Cruz) (rabbit, 1∶2000, Santa Cruz), Carbonic Anhydrase IV (CA4) (goat, 1∶25, R&D systems), Prox1 (rabbit, 1∶50, Abcam), and Cytokeratin 8 (CK8) (rat, 1∶50, Hybridoma Bank). All immunohistochemistry was visualized on a Nikon Eclipse 80 i fluorescence microscope. For stereomicroscope observations, tongues were visualized on an Olympus MVX10 fluorescent dissecting microscope. All images were analyzed using Adobe Photoshop (sizing, brightness or contrast adjustments, etc.). Brightness and contrast was adjusted linearly across the entirety of each image.

### Statistics

Data are shown as mean ± SD. Paired data were evaluated using Student’s *t*-test. *P* values less than 0.05 were considered statistically significant.

## Results

### *Lgr5* Expression in Taste Papillae

**Figure 1:**
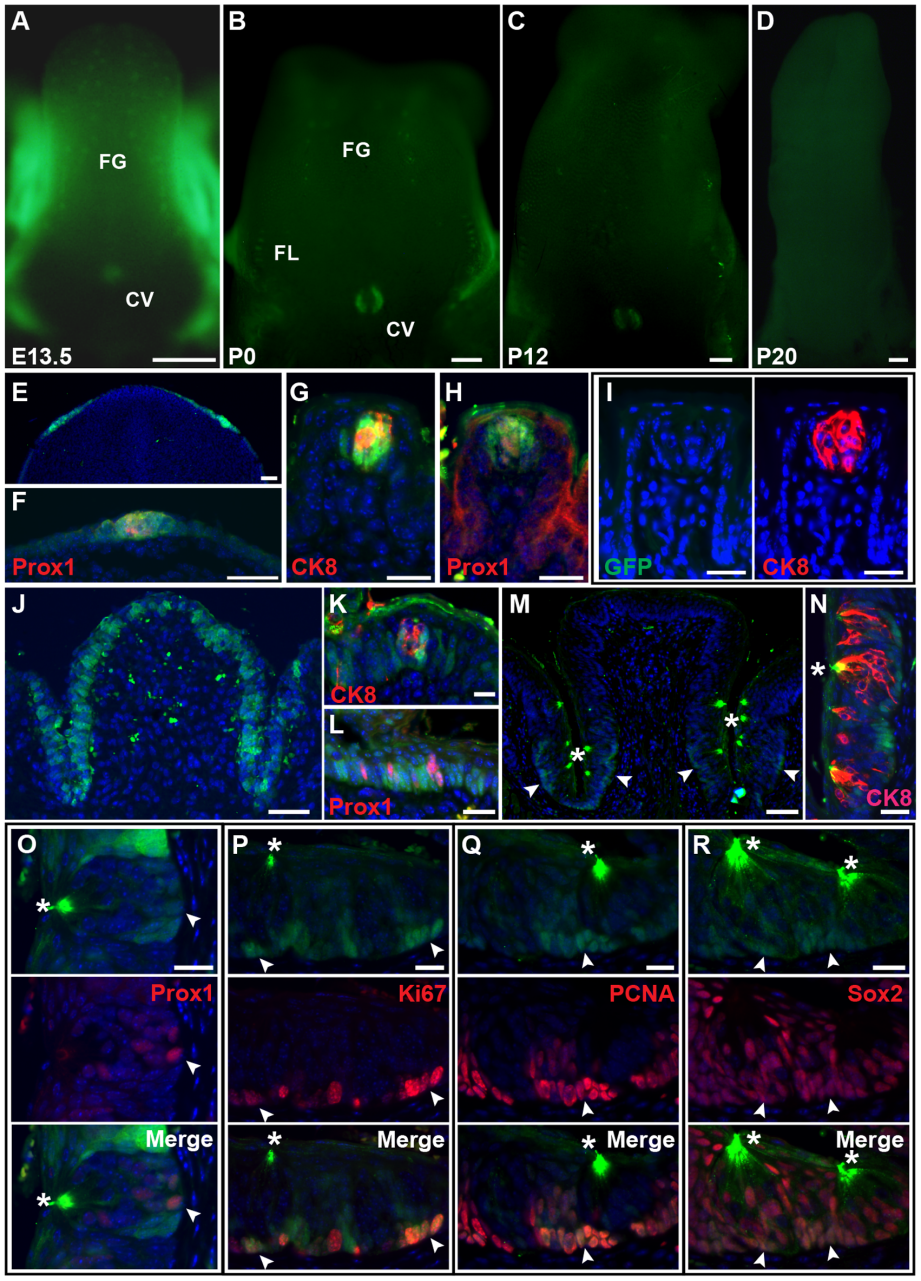
*Lgr5* expression during taste papillae development. (A–D) Whole mount EGFP fluorescence of *Lgr5^EGFP-ERCre/+^* mice tongues at E13.5 (A), P0 (B), P12 (C) and P20 (D). FG, fungiform papillae; FL, foliate papillae; CV, circumvallate papillae. (E–R) Tongue sections of *Lgr5^EGFP-ERCre/+^* mice. (E, F) GFP staining of *Lgr5^EGFP-ERCre/+^* tongue at E13.5, with Prox1, a placode marker (F). (G, H) GFP staining of *Lgr5^EGFP-ERCre/+^* FG papillae at P0.5, with CK8 (G) and Prox1 (H) co-staining. (I) GFP (left panel) and CK8 (right panel) staining on the same section from *Lgr5^EGFP-ERCre/+^* FG papillae at P50, demonstrating that *Lgr5* is not expressed within adult FG papillae. (J–L) GFP staining of *Lgr5^EGFP-ERCre/+^* CV papillae at P0.5, with CK8 (K) and Prox1 (L) co-staining. (M–R) GFP staining of *Lgr5^EGFP-ERCre/+^* CV papillae at P50, with indicated markers (N–R). Non-specific signal is detected at apical tips of taste receptor cells and surface layers of the tongue (asterisks; see Fig. S1). (M) *Lgr5* is locally expressed in basal layers in trenches of the CV papillae (arrowheads), but not outside CV papillae. (O–R) Sections were stained with GFP (upper panel) and indicated antibodies (middle panel). Merged images are shown in the lower panels. Arrowheads point to double positive cells. Scale bars = 20 µm (G–I, K, L, N–R), 50 µm (E, F, J, M), and 500 µm (A–D).

To identify *Lgr5* cells in tongue epithelium, we used *Lgr5-EGFP-IRES-ERCre* knock-in mice (*Lgr5^EGFP-ERCre/+^*), which express both EGFP and Cre-ERT2 fusion protein from the endogenous *Lgr5* locus [12]. EGFP fluorescence was detected in the region of the developing taste papillae from mid gestation through the first few weeks after birth. However, expression gradually declined and became undetectable from intact tongue specimens by postnatal day 20 (P20) (Fig. 1A–D). *Lgr5* was broadly expressed by tongue epithelium at embryonic day 13.5 (E13.5), and expression was greater in the Prox1^+^ placode than the surrounding tongue epithelium (Fig. 1E, F). *Lgr5* was also expressed by the early postnatal fungiform (FG) taste papillae of the anterior tongue. Expression overlapped with that of CK8 and Prox1 in the taste bud and was also evident in the surrounding epithelium (Fig. 1G, H). However, *Lgr5* expression was absent in adult mature FG papillae (Fig. 1I).

In contrast, *Lgr5* expression could be detected in developing circumvallate (CV) and foliate (FL) taste bud papillae of the posterior tongue as well as in adult mature stages (Fig. 1J–N and data not shown). At 0.5 day after birth (P0.5), CV papillae demonstrated shallow epithelial trenches. *Lgr5* was expressed within the papillae epithelium as well as CK8^+^ and Prox1^+^ immature taste bud cells. However, it was absent from the epithelium surrounding the CV papillae (Fig. 1J–L). As the CV trenches deepened and the number of taste buds increased with age [21], *Lgr5* expression gradually decreased and localized to the local epithelium surrounding adult taste buds (Fig. 1M and S1). Expression was most pronounced within the basal epithelium immediately surrounding taste buds (Fig. 1N *). Lgr5* expression was also detected within Prox1^+^ type IV taste bud basal cells (Fig. 1O). Most adult *Lgr5^+^* cells were cycling cells (Ki67^+^/PCNA^+^) (Fig. 1P, Q) and coexpressed Sox2 (Fig. 1R), a marker of taste bud progenitors located outside the taste bud itself [9], [10]. These data suggest that *Lgr5^+^* cells have important roles in the development and maintenance of taste buds.

### *Lgr5* Expression Defines Taste Bud Progenitor Cells

**Figure 2:**
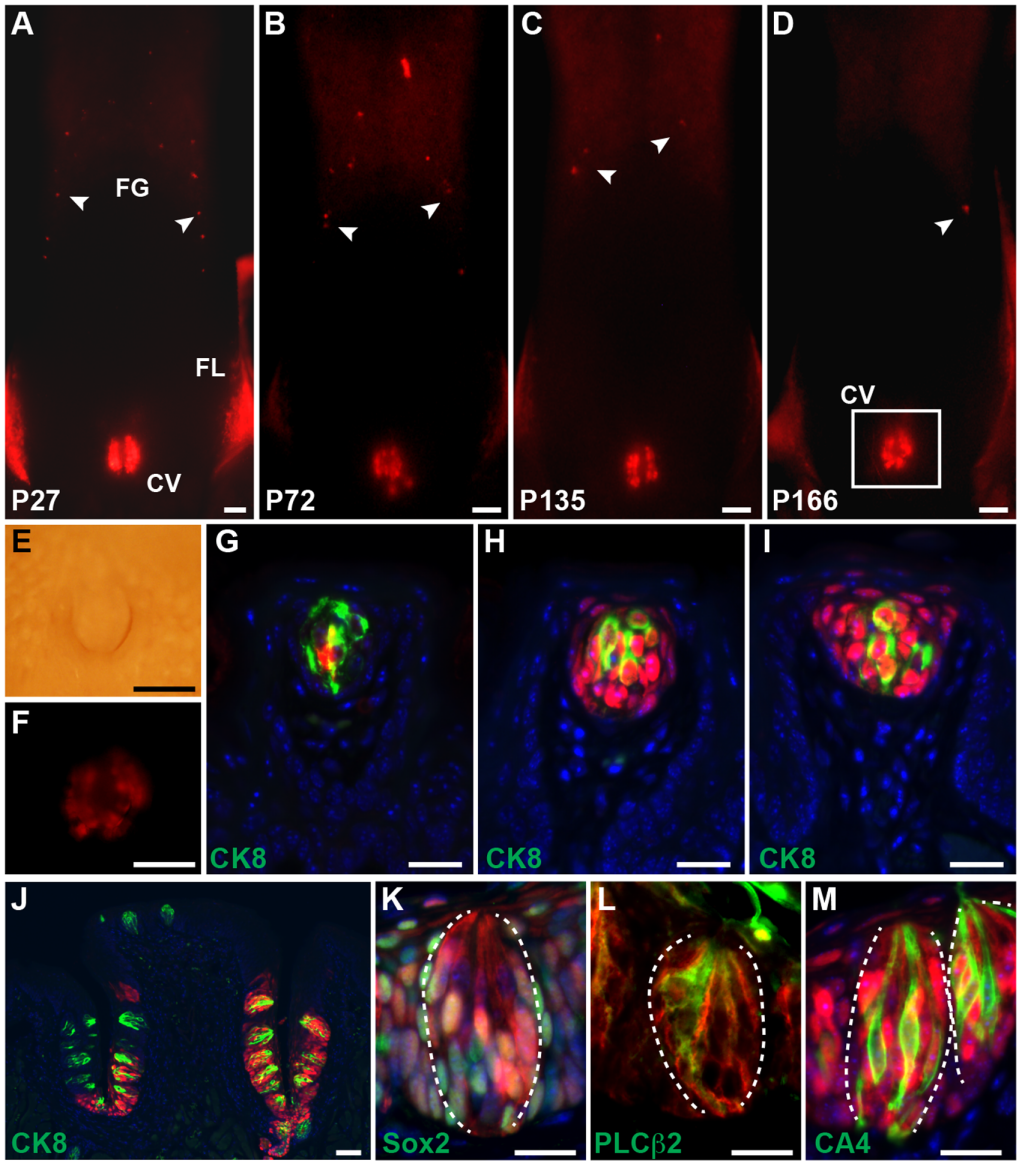
Lineage tracing of neonatal *Lgr5* cells. (A–D) Whole mount tdTomato expression of *Lgr5^EGFP-ERCre/+^*;*R26^Tom/+^* mice tongues, which were pulsed with tamoxifen once at P1 and sacrificed at P27 (A), P72 (B), P135 (C) and P166 (D). The number of labeled anterior taste papillae (arrowheads) gradually decreased and were rarely detected by 5 months after treatment, while posterior taste papillae remained strongly labeled throughout this period. (E, F) Magnified brightfield (E) and fluorescent (F) images of boxed area (D). (G–I) Double staining of *Lgr5^EGFP-ERCre/+^* mice FG papillae for tdTomato/RFP and CK8, which were pulsed with tamoxifen once at P1 and sacrificed at P27 (G, H) and P166 (I). (G, H) Most labeled FG papillae had *Lgr5*-derivatives only within taste buds (G), while a small population of FG papillae were composed of tdTomato-positive taste buds and surrounding epithelium (H). (I) FG papillae contained *Lgr5*-derivatives both within taste buds and in the surrounding epithelium after long term lineage-tracing. (J–M) TdTomato/RFP staining of *Lgr5^EGFP-ERCre/+^*;*R26^Tom/+^* mice CV papillae, which were pulsed with tamoxifen once at P1 and sacrificed at P166, with indicated markers. (K–M) Taste buds are outlined by dotted white line. Scale bars = 20 µm (G–I, K–M), 50 µm (J), and 500 µm (A–F).

To track the fate of *Lgr5^+^* cells, we crossed *Lgr5^EGFP-ERCre/+^* mice with *R26^Tom/+^* indicator mice [20], in which tdTomato expression can be induced by Cre-mediated recombination. Tamoxifen was injected intraperitoneally to activate Cre-recombinase at two different times: P1 and P50. When *Lgr5-*expressing cells were labeled at P1, both anterior and posterior taste papillae were labeled 1 month after the treatment, and could still be detected at 5 months (Fig. 2A–F). One month after treatment, 87% (75/86) of labeled FG papillae demonstrated tdTomato expression confined to the taste buds (Fig. 2G), while 13% (11/86) had expression in taste buds and the surrounding epithelium (Fig. 2H). Six months after treatment, all *Lgr5*-derived FG papillae (n = 7) revealed tdTomato expression within taste buds and the surrounding epithelium (Fig. 2I). This pattern of expression and labeling suggests that long-lived or self-renewing *Lgr5^+^* cells reside in the surrounding epithelium rather than in the taste bud. On the other hand, posterior CV papillae 1 and 5 months after treatment displayed similar tdTomato expression patterns: both taste buds and the surrounding epithelium, including type I (Sox2^+^), type II (PLC β2^+^) and type III (CA4^+^) taste cells, contained derivatives of *Lgr5^+^* cells (Fig 2J–M and S2).

**Figure 3:**
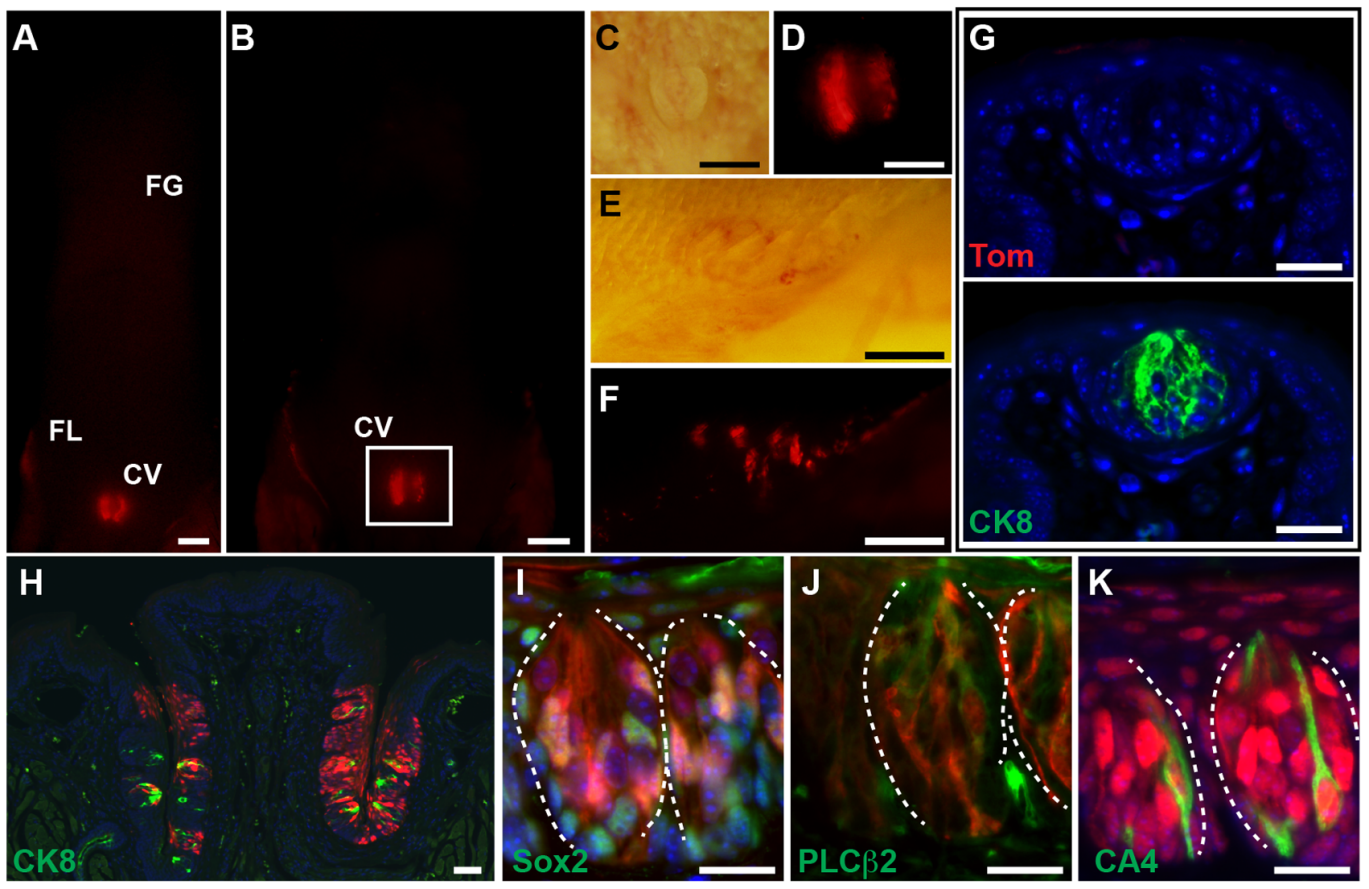
Lineage tracing of adult *Lgr5* cells. (A–F) *Lgr5^EGFP-ERCre/+^*;*R26^Tom/+^* mice were pulsed with tamoxifen at P50–54 and sacrificed at P55 (A) and 15 months later (B–F). (A, B) Whole mount tdTomato expression. Labeled anterior FG papillae were not detected at any time point. (C, D) Magnified brightfield (C) and fluorescent (D) images of boxed area (B). (E, F) Brightfield (E) and fluorescent (F) FL papillae images from the same animal shown in (B). (G–K) TdTomato/RFP immunohistochemistry of FG papillae (G) and CV papillae (H–K). [(G) taken from (A) and (H–K) from (B)] (G) TdTomato/RFP (upper panel) and CK8 (lower panel) staining of FG papillae on the same section, demonstrating that *Lgr5*-derived cells were absent within FG papillae. (H–K) RFP co-staining with indicated markers of the CV papillae. Taste buds are outlined by dotted white line. Scale bars = 20 µm (G, I–K), 50 µm (H), and 500 µm (A–F).

In a second series of experiments, adult *Lgr5^EGFP-ERCre/+^*;*R26^Tom/+^* mice were pulsed with tamoxifen for 5 consecutive days (P50–54). Only posterior taste papillae were labeled, and fated cells remained present 15 months after tamoxifen induction (Fig. 3A–F and data not shown). *Lgr5-*derivatives were not detected at any time in the FG papillae (Fig. 3G). However, *Lgr5*-derivatives marked every type of taste receptor cells and surrounding epithelium in posterior taste papillae 15 months after tamoxifen (Fig. 3H–K). No ectopic reporter activity was detected in uninduced control mice (*Lgr5^EGFP-ERCre/+^*;*R26^Tom/+^*, Fig. S3). Taken together, these results suggest that neonatal *Lgr5* expression defines progenitor cell populations in both anterior and posterior taste papillae that can give rise to taste receptor cells and/or the surrounding keratinocytes, while adult *Lgr5* expression marks progenitor cells of the posterior taste papillae only.

### *Shh* Cells of Adult Taste Papillae are a Transient Precursor of Taste Bud Cells

**Figure 4:**
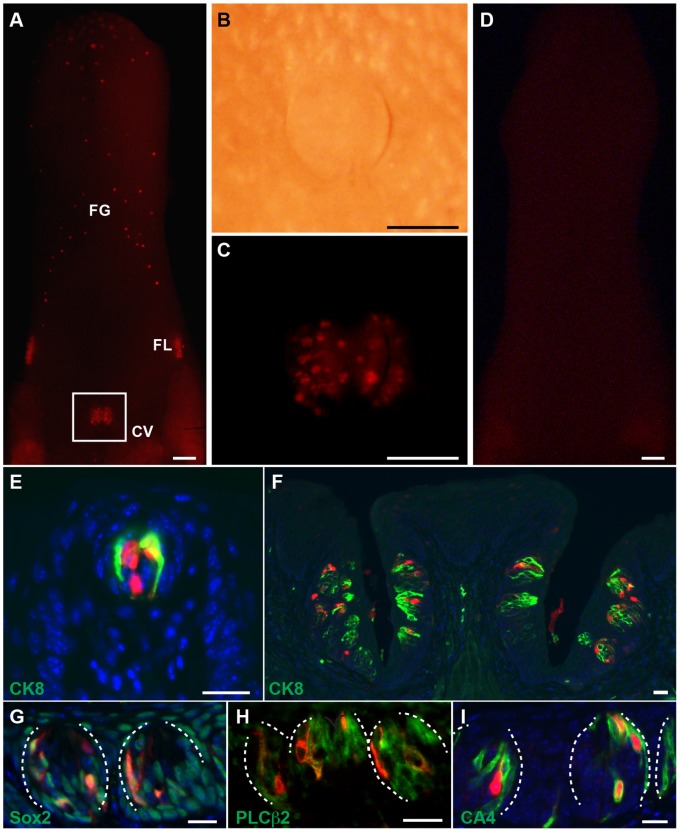
Lineage tracing of adult *Shh^+^* cells. (A–D) *Shh^ERCre/+^*;*R26^Tom/+^* mice were pulsed with tamoxifen at P50–54 and sacrificed at P80 (A–C) and P136 (D). TdTomato expression in the tongue (A, C, D), and the magnified brightfield (B) and fluorescent (C) images of boxed area (A) are shown, demonstrating that labeled *Shh*-derivatives disappeared over the next 3 months. (E–I) TdTomato/RFP stained sections from *Shh^ERCre/+^*;*R26^Tom/+^* mice FG (E) and CV (F–I) papillae shown in Fig. 4A–C, with indicated markers. (G–I) Taste bud is outlined by dotted white line. Scale bars = 20 µm (E, G–I), 50 µm (F), and 500 µm (A–D).

Adult *Shh* ^+^ type IV cells have been suggested to be a transient precursor cell population of adult taste buds [2], though this has not been experimentally tested. Therefore, we conducted lineage tracing experiments using adult *Shh^ERCre/+^*;*R26^Tom/+^* mice (tamoxifen pulse P50–54) [19]. *Lgr5* is also expressed by occasional intragemmal type IV cells (Fig. 1O). One month after tamoxifen treatment, *Shh*-derived cells were evident in most tongue taste papillae (Fig. 4A–C) but disappeared over the subsequent 2 months (Fig. 4D). *Shh*-derived cells were located only within taste buds but not the surrounding epithelium (Fig. 4E, F), and *Shh* ^+^ cells gave rise to all taste receptor cell types (Fig. 4G–I). Derivatives could not be detected in any taste papillae 3 months after induction (data not shown). Taste receptor cells are replaced approximately every 8–22 days (depending on the exact cell type) [3] and *Lgr5^+^* cells can contribute to long-term maintenance of taste receptor cells. Therefore, we conclude that the type IV cells marked by *Shh*-expression are a transient precursor cell population of adult taste buds but not a long-term stem cell population. Rather, long-term maintenance is dependent upon an *Lgr5^+^, Shh^-^* population. Further definition of the heterogeneous population of type IV cells remains to be elucidated. In addition, *Lgr5*-derived cells mark extragemmal epithelial cells and are not formed from *Shh^+^* cells, suggesting the existence of an extragemmal adult taste bud stem cell niche.

### *Lgr5* Cells Participate in Regeneration of Taste Buds

**Figure 5:**
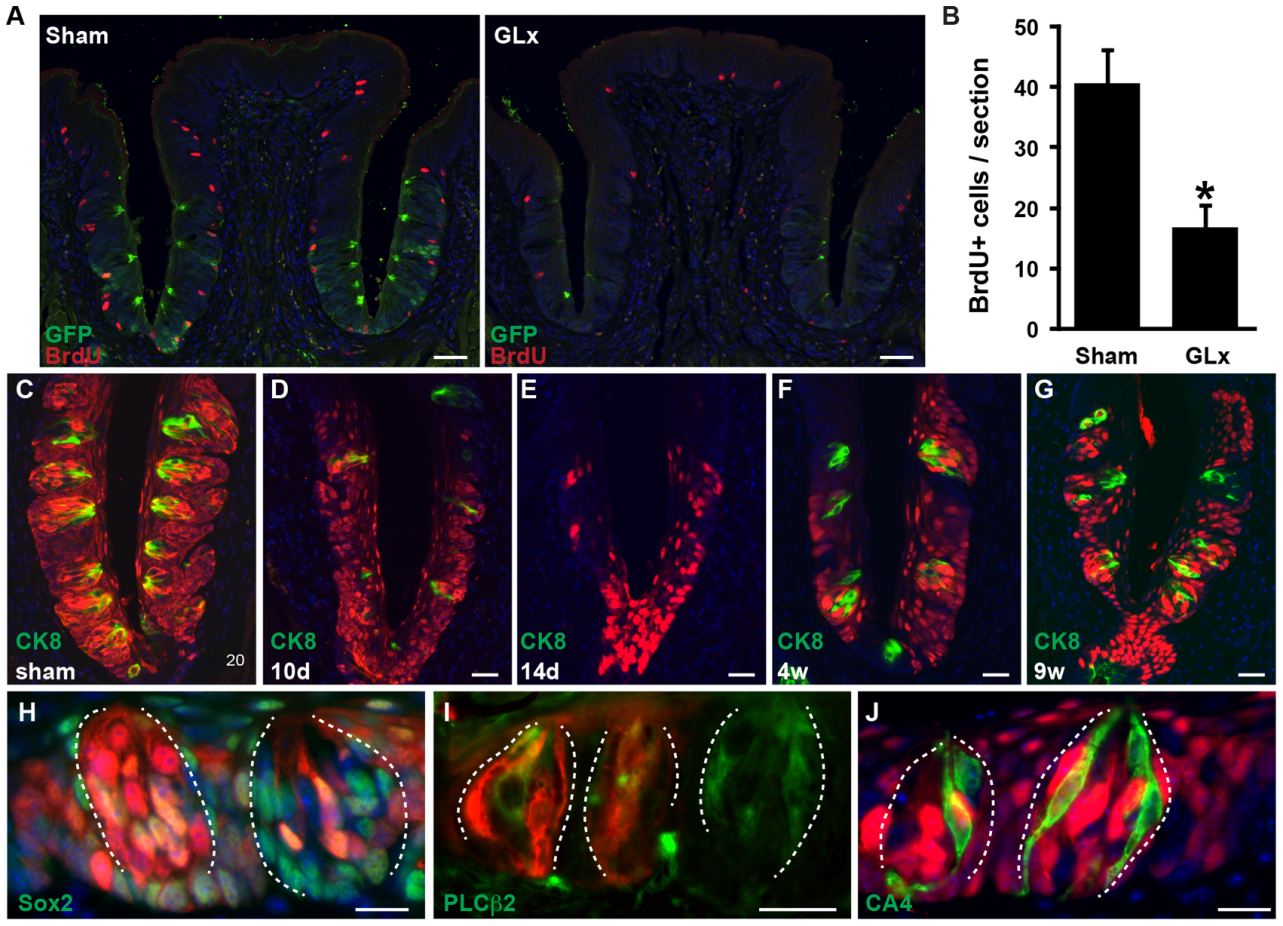
*Lgr5* cells can contribute to taste bud regeneration after GLx. *Lgr5^EGFP-ERCre/+^*;*R26^Tom/+^* mice which had been previously treated with tamoxifen at P50–54, were subjected to GLx or sham operation at P68. (A, B) *In vivo* BrdU incorporation in CV papillae. BrdU was administered 2 h prior to sacrifice 2 days after the procedure. (A) Double staining of CV papillae with GFP and BrdU. (B) Quantification of BrdU-positive cells in CV papillae. n = 4, each group. *P<0.001 vs. sham. (C–G) Double staining of *Lgr5^EGFP-ERCre/+^* mice CV papillae for tdTomato/RFP and CK8 at indicated time points after the procedure, demonstrating that *Lgr5*-derivatives survive GLx-induced injury and contribute to regenerated taste buds. (H–J) TdTomato/RFP staining of CV papillae 4 weeks after GLx, with indicated markers. (H–J) Taste buds are outlined by dotted white line. Scale bars = 20 µm (C–J), and 50 µm (A).

We sought to determine if *Lgr5^+^* cells could reconstitute taste buds after injury. Transection of the glossopharyngeal nerves bilaterally (GLx) results in degeneration of posterior taste papillae followed by regeneration over the ensuing several weeks [2], [22]. We pulsed *Lgr5^EGFP-ERCre/+^*;*R26^Tom/+^* mice with tamoxifen at P50–54 and performed GLx 2 weeks later (Fig. S4). Two days after injury, *Lgr5* expression and cell proliferation (measured by BrdU incorporation and usually robust in the epithelial compartment) was markedly reduced (Fig. 5A, B). As expected, CK8*^+^* taste bud cells gradually disappeared and were no longer detected by 14 days after GLx (Fig. 5C–E) [22]. Notably, many *Lgr5*-derivatives within the local epithelium survived GLx-induced injury (Fig. 5E). Regenerated taste buds were apparent four weeks after GLx (Fig. 5F, G and S4), and all three types of taste receptor cells derived from *Lgr5*-expressing progenitors (Fig. 5H–J). Nine weeks after GLx, *Lgr5*-derivatives were still present in all taste bud cell types and the surrounding local epithelium (Fig. 5G and data not shown), suggesting that adult *Lgr5^+^* cells surrounding taste buds in posterior papillae are a progenitor population contributing to taste bud regeneration upon injury.

## Discussion

In this report, we demonstrate that *Lgr5-*expressing cells in the newborn give rise to taste buds in both the anterior and the posterior regions of the tongue. *Lgr5^+^* cells can contribute to all the lineages of the taste bud, including various receptor cell types and local keratinocytes. In the adult, *Lgr5* is expressed by the epithelium surrounding adult posterior taste buds and by some intragemmal type IV basal cells. Adult posterior *Lgr5^+^* cells survive damage following glossopharyngeal nerve injury and contribute to regenerating taste buds. *Lgr5* is not expressed in anterior taste buds in the adult.

Taken together, our data is most consistent with a model in which extragemmal *Lgr5* expression identifies a posterior taste bud stem/progenitor cell population. Data to support this model includes the finding that lineage tracing of *Shh*-expressing cells, which is confined to the intragemmal region and does not provide long-term labeling of regenerated taste buds. Derivatives of *Lgr5^+^* cells were found in the adjacent extragemmal epithelium in every instance of long-term *Lgr5* lineage tracing in which taste buds were labeled. Labeling confined to the intragemmal taste bud was observed only after short chase periods. Hence, long-term repopulating cells are likely to be confined to the extragemmal domain.

*Lgr5*-expressing cells function as taste bud progenitor cells throughout the tongue during early postnatal life, but *Lgr5* expression is lost in adult anterior taste papillae. Therefore *Lgr5-*expression does not mark a progenitor cell in that location. Thus, further studies are necessary to fully characterize stem/progenitor cells in the full range of taste receptors. We recently demonstrated that *Hopx* expression identifies multiple adult epithelial stem cell populations with subtle differences from *Lgr5^+^* cells with regard to the ability of expressing cells to proliferate and regenerate under physiological conditions [23], [24]. *Hopx* is strongly expressed by slowly cycling, BrdU-retaining stem cells in the hair follicle bulge and intestinal crypt. In taste papillae, *Hopx* is absent from basal epithelium surrounding taste buds, but present elsewhere within basal cells of tongue epithelium (Epstein laboratory, unpublished data). Combinations of markers such as *Lgr5*, *Hopx, Shh* and others may provide tools to identify and to more precisely define progenitor cells that contribute to taste bud formation and regeneration during embryogenesis and adulthood.

In summary, our studies identify *Lgr5* as a marker of progenitor cells that will contribute to multiple taste bud populations during development. In the adult, *Lgr5*-expressing taste bud progenitors are restricted to the posterior tongue, where they are capable of regenerating all the cell types of the mature taste bud after injury induced by denervation. Further definition of the signals that mediate activation of taste bud stem cells may be useful therapeutic targets to hasten recovery of taste and appetite in patients suffering from iatrogenic loss of these faculties.

## Supporting Information

Figure S1 **GFP staining of adult wild-type mouse CV papillae.** Non-specific signals are detected at apical tips of taste bud cells (asterisks) and surface layers of the tongue in wild type mice. Scale bars = 50 µm.
(TIF)

Figure S2 **Sox2 within taste buds mark type I taste receptor cells.** Double staining of Sox2 of taste papillae with type II (PLC β2, A) and III (CA4, B)-specific markers. Sox2 expression is detected in both intragemmal and extragemmal epithelial cells, and intragemmal Sox2 is expressed in non type II/III cells. The taste bud is outlined by dotted white line. Scale bars = 20 µm.
(TIF)

Figure S3 **Control staining of uninduced** ***Lgr5^EGFP-ERCre/+^*** **;** ***R26^Tom/+^*** **mice.** TdTomato/RFP and CK8 staining of the CV (A) and FG papillae (B) from an adult *Lgr5^EGFP-ERCre/+^*;*R26^Tom/+^* mouse that has not been injected with tamoxifen, demonstrating no ectopic expression of tdTomato/RFP. Scale bars = 50 µm.
(TIF)

Figure S4 **Taste bud regeneration after transection of bilateral glossopharyngeal nerves (GLx).** Representative images of hematoxylin/eosin-stained sections of CV papillae at indicated time points after GLx. Taste buds gradually disappear by 14 days after GLx followed by the appearance of normal-looking taste buds (3 w, 4 w, arrowheads). Numerous regenerated taste buds are present 9 weeks after GLx. Scale bars = 50 µm.
(TIF)
