# Supplementary figures and images for: Lgr5 Identifies Progenitor Cells Capable of Taste Bud Regeneration after Injury
Source: PLoS One. 2013 Jun 18;8(6):e66314. doi: 10.1371/journal.pone.0066314 (PMC3688887; doi:10.1371/journal.pone.0066314)

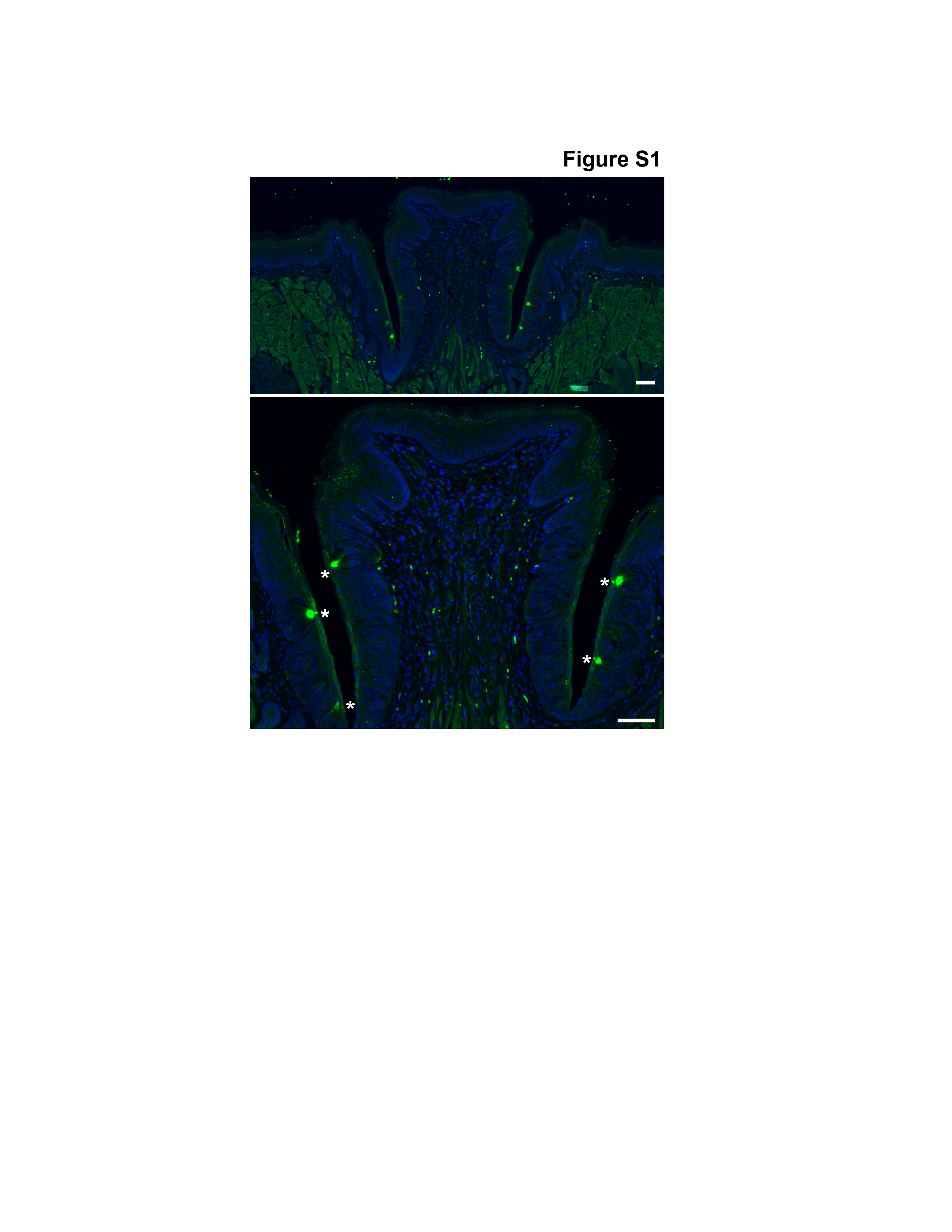

Supplement: Figure S1 — GFP staining of adult wild-type mouse CV papillae. Non-specific signals are detected at apical tips of taste bud cells (asterisks) and surface layers of the tongue in wild type mice. Scale bars = 50 µm. (TIF) [file pone.0066314.s001.tif]

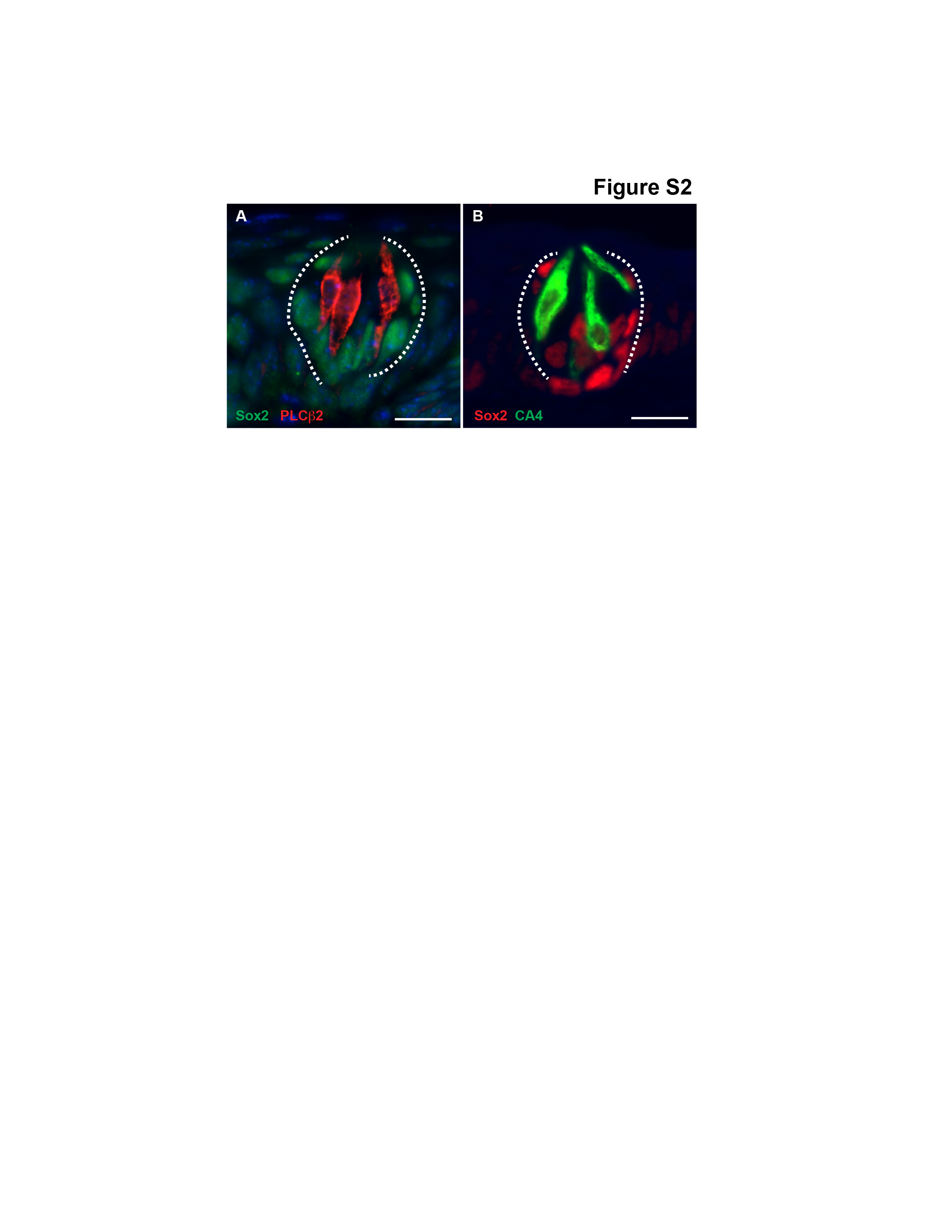

Supplement: Figure S2 — Sox2 within taste buds mark type I taste receptor cells. Double staining of Sox2 of taste papillae with type II (PLC β2, A) and III (CA4, B)-specific markers. Sox2 expression is detected in both intragemmal and extragemmal epithelial cells, and intragemmal Sox2 is expressed in non type II/III cells. The taste bud is outlined by dotted white line. Scale bars = 20 µm. (TIF) [file pone.0066314.s002.tif]

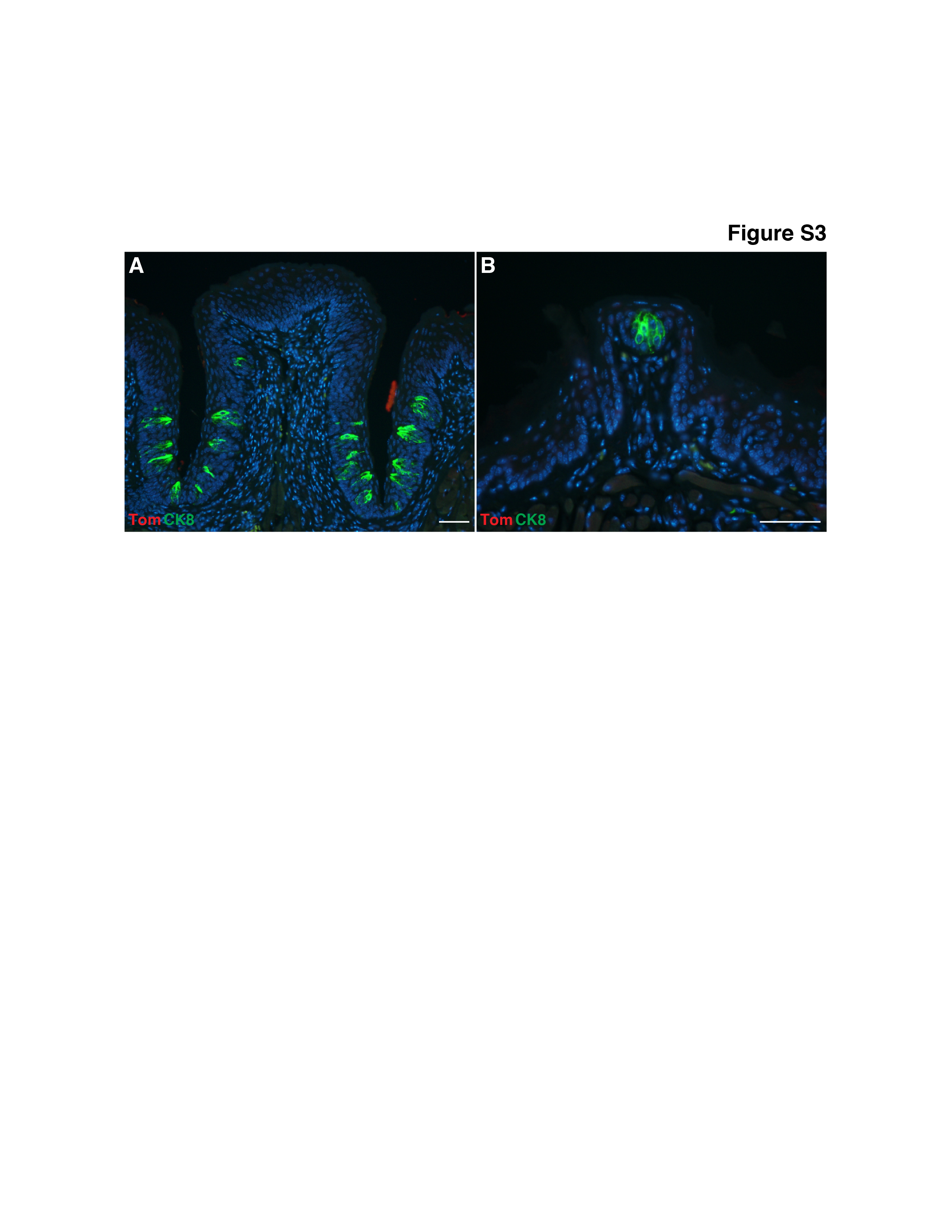

Supplement: Figure S3 — Control staining of uninduced Lgr5EGFP-ERCre/+ ; R26Tom/+ mice. TdTomato/RFP and CK8 staining of the CV (A) and FG papillae (B) from an adult Lgr5EGFP-ERCre/+;R26Tom/+ mouse that has not been injected with tamoxifen, demonstrating no ectopic expression of tdTomato/RFP. Scale bars = 50 µm. (TIF) [file pone.0066314.s003.tif]

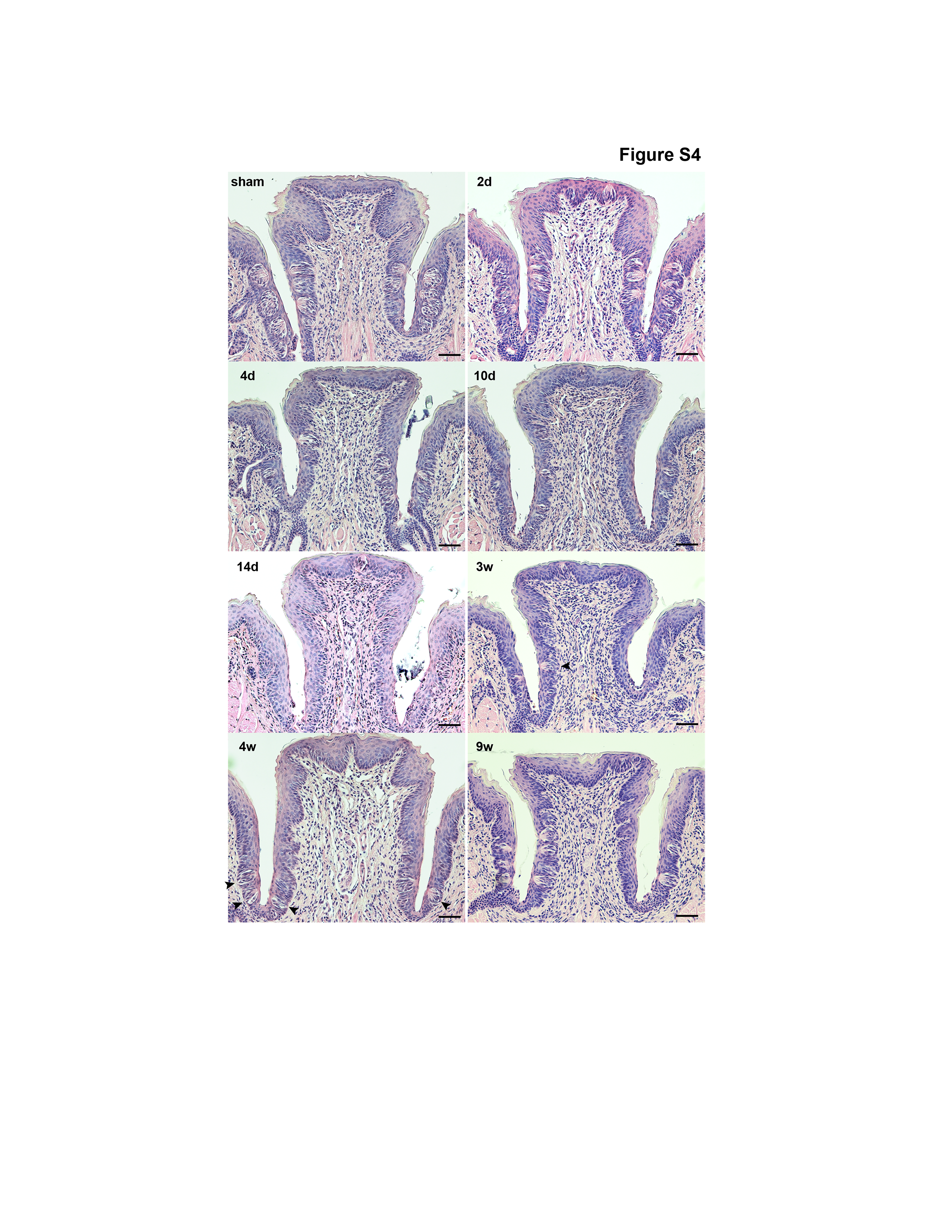

Supplement: Figure S4 — Taste bud regeneration after transection of bilateral glossopharyngeal nerves (GLx). Representative images of hematoxylin/eosin-stained sections of CV papillae at indicated time points after GLx. Taste buds gradually disappear by 14 days after GLx followed by the appearance of normal-looking taste buds (3 w, 4 w, arrowheads). Numerous regenerated taste buds are present 9 weeks after GLx. Scale bars = 50 µm. (TIF) [file pone.0066314.s004.tif]
